# Effects of Aminoguanidine on Lipid and Protein Oxidation in Diabetic Rat Kidneys

**DOI:** 10.1080/15604280214487

**Published:** 2002

**Authors:** Dilek Gogas Yavuz, Belgin Küçükkaya, H. önder Ersöz, A. Süha Yalçin, Kaya Emerk, Sema Akalin

**Affiliations:** 1 Department of Internal Medicine Section of Endocrinology and Metabolism School of Medicine Marmara University Istanbul Turkey; 2 Department of Biochemistry School of Medicine Marmara University Istanbul Turkey; 3 Moda Cad. Sakızgulu Sok. No:1-3/15, Kadıkoy Istanbul 81030 Turkey

## Abstract

Nonenzymatic glycation of tissue and plasma proteins may stimulate the production of oxidant and carbonyl stress in diabetes. The aim of this study was to evaluate the effects of aminoguanidine (AG) on lipid peroxidation, protein oxidation and nitric oxide (NO) release in diabetic rat kidneys. After induction of diabetes with streptozotocin, female Wistar rats were divided into 2 groups. Group DAG (n=9) rats were given AG hydrogen carbonate (1 g/L) in drinking water and group D (n=8) was diabetic control rats given only tap water. Group H (n=8) was followed as healthy controls. At the end of an 8 week period, NO release, lipid and protein oxidation were determined in kidney tissues. NO release was significantly lower in diabetic rats compared with healthy controls (p<0.05). Lipid peroxidation was significantly high in group D (3.9 ± 0.3 nmol MDA/g tissue) compared with the group DAG (2.6 ± 0.1 nmol MDA/g tissue, p<0.01) and group H (2.4 ± 0.2 nmol MDA/g tissue). Protein oxidation was significantly higher in diabetics than healthy controls (563.8 ± 23.9, 655.8 ± 7.2 , 431.5 ± 8.8 mmol carbonyl / g tissue for group DAG, D and H, respectively, p< 0.05). A positive correlation between albuminuria and thiobarbituric acid reactive substance (TBARS) levels (r= 0.54,p<0.005) and carbonyl content (r=0.70, p<0.0005) in kidney homogenate were observed.

Although AG treatment had no effect on NO release, it significantly decreased lipid peroxidation in diabetic rat cortices. Consequently increased lipid peroxidation -as well as- protein oxidation could be involved in the pathogenesis of diabetic albuminuria.


*Int. Jnl. Experimental Diab. Res.*, 3:145-151, 2002
© 2002 Taylor and Francis
1560-4284/02 $12.00 + .00

### Effects of Aminoguanidine on Lipid and Protein Oxidation in Diabetic Rat Kidneys

DILEK GOGAS YAVUZ*, BELGIN KÜÇÜKKAYA**, H.ÖNDER ERSÖZ*, A.SÜHA YALÇIN**, KAYA EMERK**, SEMA AKALIN*

*Department of Internal Medicine Section of Endocrinology and Metabolism and
** Department of Biochemistry
School of Medicine, Marmara University, Istanbul TURKEY

*Received: June 14, 2001; In final form: November 20, 2001*

**Nonenzymatic glycation of tissue and plasma proteins may stimulate the production of oxidant and carbonyl stress in diabetes. The aim of this study was to evaluate the effects of aminoguanidine (AG) on lipid peroxidation, protein oxidation and nitric oxide (NO) release in diabetic rat kidneys. After induction of diabetes with streptozotocin, female Wistar rats were divided into 2 groups. Group DAG (n=9) rats were given AG hydrogen carbonate (1 g/L) in drinking water and group D (n=8) was diabetic control rats given only tap water. Group H (n=8) was followed as healthy controls. At the end of an 8 week period, NO release, lipid and protein oxidation were determined in kidney tissues. NO release was significantly lower in diabetic rats compared with healthy controls ($p<0.05$). Lipid peroxidation was significantly high in group D (3.9 ± 0.3 nmol MDA/g tissue) compared with the group DAG (2.6 ± 0.1 nmol MDA/g tissue, $p<0.01$) and group H (2.4 ± 0.2 nmol MDA/g tissue). Protein oxidation was significantly higher in diabetics than healthy controls (563.8 ± 23.9, 655.8 ± 7.2 , 431.5 ± 8.8 mmol carbonyl / g tissue for group DAG, D and H, respectively, $p< 0.05$). A positive correlation between albuminuria and thiobarbituric acid reactive substance (TBARS) levels (r=**

*Corresponding author: Moda Cad. Sakızgulu Sok. No:1-3/15, Kadıkoy,81030 Istanbul,Turkey; Tel: 90 216 4490347; Fax: 90 216 4490347; e-mail: dyavuz@turk.net

**0.54,p<0.005) and carbonyl content (r=0.70, p<0.0005) in kidney homogenate were observed.**

**Although AG treatment had no effect on NO release, it significantly decreased lipid peroxidation in diabetic rat cortices. Consequently increased lipid peroxidation -as well as- protein oxidation could be involved in the pathogenesis of diabetic albuminuria.**

### INTRODUCTION

Nonenzymatic glycation of long lived structural proteins is one of the mechanisms that is involved in the pathogenesis of diabetic nephropathy [1-4]. Glycation of tissue and plasma proteins may stimulate the production of oxidant and carbonyl stress in diabetes [5,6]. Increased lipid and protein oxidation are thought to trigger diabetic tissue damage [7,8]. Oxidized lipids may in turn stimulate oxidative reactions of sugars, enhancing damage to both lipids and proteins [7-9].

Aminoguanidine, (AG) an inhibitor of advanced glycation, was shown to prevent lipid peroxidation in human plasma and red blood cell membranes [10] as well as rat tissues [11]. It is also known to be a selective inhibitor of inducible nitric oxide synthase [12].

Increased glucose and free fatty acids may stimulate endothelial cells and macrophages to secrete nitric oxide (NO) and super oxide which is increased peroxynitrite formation in diabetic milieu [13]. It is not clear whether inhibition of NO production by AG influences lipid peroxidation in diabetic tissues.

Carbonyl groups are the end products of protein oxidation. Their levels in tissues and plasma serve as relatively stable markers of oxidative damage [14,15]. Reactive carbonyl compounds and residual carbonyl groups of modified proteins react covalently with matrix tissue proteins and alter their structure and function [9,16]. Carbonyl modification of proteins by auto oxidation of sugars is thought to be associated with kidney damage in diabetes [17]. Cross talk between oxidative stress, protein oxidation and NO metabolism seems to be complex in diabetic milieu and the effects of advanced glycation inhibition in this setting remains to be determined.

The aim of this study was to evaluate the effects of aminoguanidine on NO release, lipid and protein oxidation in diabetic kidney tissues. The relationship between urinary albumin excretion and lipid peroxidation , protein oxidation were also evaluated.

### MATERIALS AND METHODS

Twenty-five ten-week-old female Wistar rats were used in the study. Seventeen rats were made diabetic by i.p injection of 65 mg/kg Streptozotocin in sodium citrate buffer pH 4.5. Eight rats receiving an equivalent amount of buffer served as healthy controls (Group H) . One week after induction of diabetes, 9 rats (Group DAG) were given aminoguanidine bicarbonate (Sigma MO,USA) ad libitum in drinking water at a concentration of 1 g/L [18]. Eight diabetic rats were followed up as diabetic controls (Group D) and given only tap water.

None of the groups received hypoglycemic agents. During the study, rats were kept in temperature (25°C) and light-(12h light,12h dark) controlled rooms and fed a standard 20% protein-containing rat chow. All experiments were conducted in accordance with internationally accepted principles for the care and use of laboratory animals and were approved by the committee for animal research of Marmara University Medical School.

At the end of the 8-week period, 24-hour urine samples were collected in metabolic cages. Blood samples were collected by cardiac puncture while rats were kept under ether anesthesia. All rats were sacrificed under ether anesthesia. Their kidneys were rapidly dissected and exposed to liquid nitrogen and immediately stored at -70 $^0$C until use.

**Determination of plasma glucose and creatinine**: Plasma glucose was measured with a colorimetric method using glucose oxidase (Boehringer Mannheim, Germany) for Hitachi 705 autoanalyser.

**Determination of urinary albumin excretion:** Urinary albumin excretion was determined by using a Titan gel electrophoresis kit from Helena Laboratories (Sunderland, UK).

**Tissue preparation**: Rat kidney cortices were dissected (40-50mg) and put into scintillation vials for NO release assay. Rat kidney cortices were also homogenized (%5, w/v) in Hank's buffer (pH 7.2) for oxidative stress parameters and sulfhydryl group assays.

**Determination of NO release**: NO release was detected by using luminol $H_2O_2$ chemiluminescence with minimal modifications described by Kikuchi et al [19]. Rat kidney cortices were placed in glass scintillation vials containing 3 ml of Hank's buffer +HEPES. Chemiluminescence probe (18 µM luminol,150 mm desferrioxamine, 10 mM $H_2O_2$, 2mM potassium carbonate) was added and chemiluminescence measurements were recorded for 5 min at 30 sec intervals. Area under the curve (AUC) was calculated for each experiment (cpm/mg tissue for 5 min).

**Determination of lipid peroxidation**: Kidney cortex homogenates (5%,w/v) were mixed with an equal volume of ice cold 10% trichloroacetic acid (TCA). After centrifugation, a volume of the supernatant was added to an equal volume of 0.67 % thiobarbituric acid (TBA) and the mixture was kept in a boiling water bath for 15 min. Samples were cooled to room temperature and absorbance at 532 nm were recorded. The results were expressed as MDA equivalents using a molar extinction coefficient of 1.56 x$10^5 M^{-1} cm^{-1}$ [20].

**Determination of tissue protein oxidation**: Carbonyl contents of proteins were determined by a modification of the procedure described by Levine et al [21]. Results were expressed as nanomoles per gram tissue using a molar extinction coefficient of 22 000 $M^{-1} cm^{-1}$.

**Statistical analyses** were performed with an IBM compatible PC using Instat III program. Kruskall Wallis ANOVA, Mann Whitney U tests were used for comparisons of the groups as appropriate. Spearman Rank test was used for the correlation analysis. Results are expressed as mean±SEM.

### RESULTS

Table 1 lists blood glucose and urinary albumin excretion (UAE) rates in all study groups. Diabetic rats had significantly elevated plasma glucose and urinary albumin levels compared to non-diabetic rats. Aminoguanidine therapy retarded the increase in urinary albumin excretion at 8 weeks to levels approaching but still higher than those of healthy control rats (D vs DAG $p<0.001$). Plasma glucose levels were not affected by aminoguanidine treatment in diabetic rats.

Lipid peroxidation, protein oxidation and NO release from rat kidney cortices are shown in Table 2. TBARS content of renal cortices in non-treated diabetic rats was significantly elevated compared to healthy ($p<0.05$) and AG treated diabetic rats ($p<0.05$). TBARS were higher in the renal cortices of the non-treated diabetic group while AG treated diabetic rats had similar values with healthy controls.

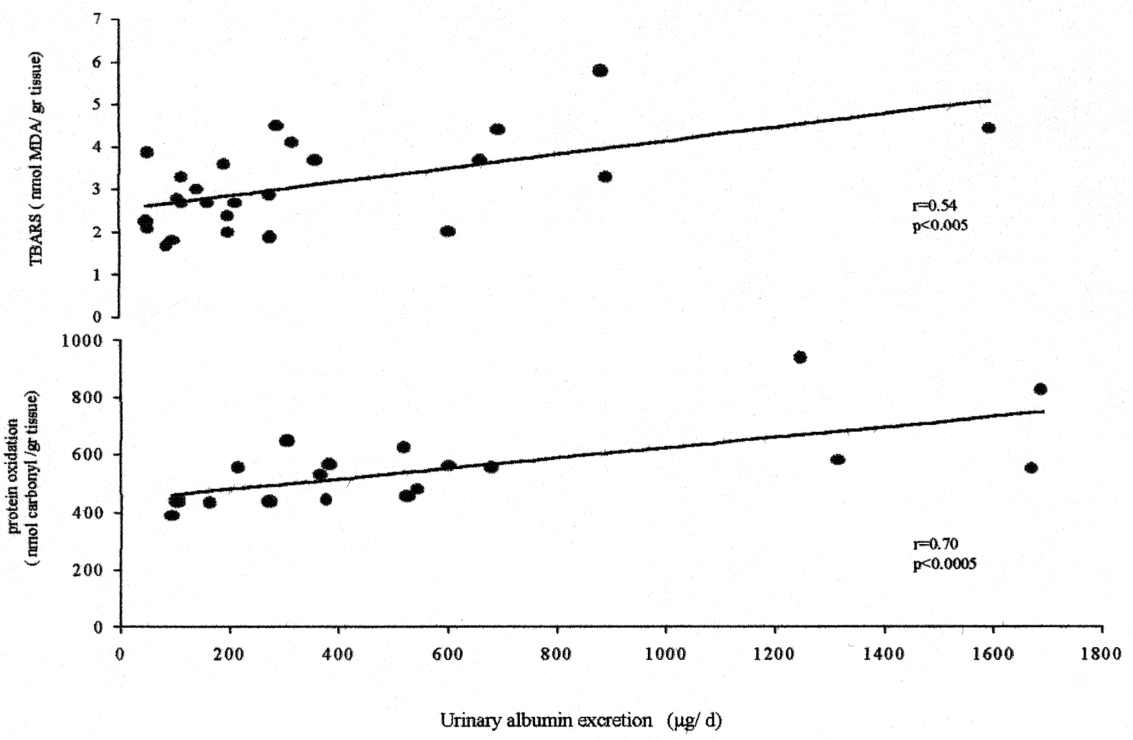

FIGURE 1
Correlation analysis of urinary albumin excretion with TBARS and carbonyl content of renal cortex tissues of all study groups.

TABLE 1 Blood glucose and urinary albumin excretion in all study groups

| | Group H | Group D | Group DAG |
|---|---|---|---|
| Glucose (mg/dl) | 86.2±17* | 466.6±125 | 389.6±128.1 |
| UAE (µg/d) | 187.5±99.4 | 1414.7±769** | 392.7±134.9 |

* p <0.001 vs other groups ,** p <0.001 vs H, p <0.05 vs DAG
UAE: Urinary albumin excretion H: Healthy control group,
D: Diabetic control group, DAG: Aminoguanidine treated diabetic group

Protein oxidation of kidney cortices could be measured only in 7 rats from the DAG group and 6 rats from each of the other groups. Diabetic rats had higher levels of protein oxidation than healthy rats ($p<0.05$). AG tended to decrease renal tissue protein oxidation levels in diabetic rats but the difference was not statistically significant compared with diabetic controls.

Urinary albumin excretion showed a significant positive correlation with renal cortex TBARS levels ($r=0.54$, $p<0.005$) and carbonyl content ($r=0.70$, $p<0.0005$) (Figure 1). There was no significant correlation between TBARS and protein oxidation levels of kidney cortices.

Non-treated diabetic rats had lower levels of NO release from kidney, compared to healthy control rat tissues and AG treatment had no

TABLE 2 Nitric oxide release, lipid peroxidation and protein oxidation measurements in rat kidney tissues

| | Group H | Group D | Group DAG |
|---|---|---|---|
| NO Release fmol.min-1 (g of kidney weight)-1 | 2440310 ± 48921 * | 497703 ± 89525 | 461844 ±174330 |
| TBARS (nmol MDA / g tissue) | 2.45 ±0.7 | 3.9 ±1.0 † | 2.6 ±0.5 |
| Protein oxidation (nmol carbonyl / g tissue) | 431.4 ±8.8 ‡ | 655.8 ±75.2 | 563.8 ±23.9 |

* $p < 0.05$ vs other groups † $p < 0.05$ vs H and group DAG, ‡ $p < 0.05$ vs other groups
TBARS: Thiobarbituric acid reactive substances, H: Healthy control group,
D: Diabetic control group, DAG: Aminoguanidine treated diabetic group significant effect on NO release.

### DISCUSSION

In this study lipid peroxidation as measured by TBARS, which is an index of malondialdehyde production, was found to be elevated in renal tissues of non-treated diabetic rats compared with healthy controls. This is in accordance with the findings of others who have shown that lipid peroxidation is increased in diabetic rats [10,22,23].

Aminoguanidine treated diabetic rats had lower lipid peroxidation in kidney cortex homogenates than non-treated diabetic rats. These results support those of Kedziora-Karnotowska et al. [10] who reported that aminoguanidine treatment attenuated the increase in MDA content and diminished activities of key antioxidant enzymes in kidney homogenates of STZ- diabetic rats.

A positive correlation between urinary albumin excretion and renal tissue MDA content might be indicating that lipid peroxidation in renal tissues could have an influence on the progression of albuminuria in diabetic rats.

Reactive oxygen species mediated reactions lead to the formation of protein carbonyl derivatives, which serves as a marker of protein damage. Reactive carbonyl compounds have been found to be present in diabetic glomerular lesions [24]. Reactive carbonyl compounds is the result of oxidative stress and could be an active contributor to pathogenesis of diabetic complications. Brownlee et al [25] had demonstrated that aminoguanidine inhibits the formation of reactive carbonyl compounds in arterial wall. In this study we observed increased protein oxidation in diabetic rat kidney tissues compared to healthy controls. Carbonyl content of kidney tissue homogenates showed a significant positive correlation with urinary albumin excretion in all study groups. This result suggests that protein oxidation might be taking a part in the pathogenesis of diabetic nephropathy. But our data failed to show a significant effect of aminoguanidine on protein oxidation in diabetic rats which could be due to small study groups.

Defective nitric oxide production is associated with diabetic nephropathy. Several studies have demonstrated that pathophysiologic and morphologic changes in diabetic nephropathy are mediated by either increase or decrease in renal NO production or activity [26,27].

$H_2O_2$ induced NO release was found to be decreased in diabetic rat kidneys in our study. This finding is in accordance with the results of Craven et al. [28] who measured basal NO production with an NO electrode in isolated glomeruli from diabetic rats and found that NO production was markedly reduced in glomeruli from two month diabetic rats. An advanced glycation inhibitor, aminoguanidine, which is also a nitric oxide synthase inhibitor, had no significant effect on $H_2O_2$ induced NO release from diabetic rat kidneys in our study. NO release showed no correlation between lipid peroxidation, protein oxidation and albuminuria. These results are in accordance with the results of Soulis et al [12], who demonstrated that aminoguanidine had no effect on inducible nitric oxide synthesis while it prevented increases in albuminuria. They conclude that the effect of aminoguanidine has been mediated predominantly by decreased AGE formation rather than via NOS inhibition [12].

Our data confirm the favorable effects of aminoguanidine in preventing the increase in albuminuria and lowering lipid peroxidation while no statistically significant effect on NO release in diabetic rats. Aminoguanidine treatment tended to lower renal cortex carbonyl content. Since the carbonyl content of kidney tissues was positively correlated with urinary albumin excretion in diabetic rats; protein oxidation could be one of the causes of albuminuria in diabetes.

In conclusion, aminoguanidine treatment reduces albuminuria and lipid peroxidation in renal cortices of diabetic rats. It may have an additional beneficial effect as an antioxidant against lipid and protein oxidation and thereby diminish diabetic albuminuria.

### REFERENCES

1. Lee H.B., Cha M.K., Song K.I., Kim J.H., Lee E.Y., Kim S.I., Kim J., Yao M.H. (1997) Pathogenic role of advanced glycosylation end products in diabetic nephropathy, *Kidney Int,* **52** (Suppl 60), S60-S65.
2. Makino H., Shikata K., Kaushiro M., Hironaka K., Yamasaki Y., Sagimato H., Ota Z., Araki N., Hariuchi S. (1996). Roles of advanced glycation end-products in the progression of diabetic nephropathy, *Nephrol Dial Transplant*, **11** (Suppl 5), 76-80.
3. Beisswenga P.J., Makita Z., Curphey J., Moore L.L., Jean S., Brinck-Johnsen T., Bucala R., Vlassara H. (1995). Formation of immunochemical advanced glycosylation end products precedes and correlates with early manifestations of renal and retinal disease in diabetes, *Diabetes*, 44, 824-829.
4. Vlassara H., Striker L.J., Teichberg S., Fuh H., Ming Li Y.M., Steffes M. (1994). Advanced glycation end products induce glomerular sclerosis and albuminuria in normal rats, *Proc Natl Acad Sci USA*, **91**, 11704-11708.
5. Laske C., Neumann A., Cunningham A.M, Nichol K., Schinzel R., Riederer P., Münch G. (1998). Cytotoxicity of advanced glycation end products is mediated by oxidative stress, *J Neurol Transm* **105**, 1005-1015.
6. Suzuki D. and Miyata T. (1999). Carbonyl stress in the pathogenesis of diabetic nephropathy, *Internal Medicine*, **38** (4), 309-314.
7. Chisolm G.M., Irwin K.C., Penn M.S. (1992). Lipoprotein oxidation and lipoprotein induced cell injury in diabetes, *Diabetes*, **41** (suppl 2), 61-66.
8. Baynes J.W. and Thorpe S.R. (1999). Role of oxidative stress in diabetic complications:a new perspective on an old paradigm, *Diabetes*, **48**, 1-9.
9. Lyons T.J., Jenkins A.J. (1997). Lipoprotein glycation and it's metabolic consequences. *Curr Opin Lipidol,* **8**:3 174-80.
10. Kedziora-Karnotowska K.Z., Luciak M., Blaszczyk J. Pawlak W. (1998). Lipid peroxidation and activities of antioxidant enzymes in erythrocytes of patients with non-insulin dependent diabetes with or without diabetic nephropathy, *Nephrol Dial Transplant*, **13**, 2829-2832.
11. Kedzora-Karnotowska K., Luciak M. (1998). Effect of aminoguanidine on lipid peroxidation and activities of antioxidant enzymes in the diabetic kidney, *Biochem Mol Biol Int*, **46**(3), 577-583.
12. Saulis T., Cooper M.E., Sastra S., Thallas V., Panagiotopoulos S., Bjerrum O.J., Jerums G. (1997). Relative contributions of advanced glycation and nitric oxide synthase inhibition to aminoguanidine mediated renoprotection in diabetic rats, *Diabetologia*, **40**, 1141-1151.
13. Traub O. and Van Bibler R. (1995). Role of nitric oxide in insulin dependent diabetes mellitus- related vascular complications, *West J Med*, **162**, 439-445.
14. Odetti P., Garibaldi S., Nobresco G., Aragno I.,Valentini S., Traverso N., Marinari U.M. (1999). Levels of carbonyl groups in plasma proteins of type 2 diabetes mellitus subjects, *Acta Diabetol*, **36**, 179-83.
15. Chevion M., Berenshtein E., Stadtman E.R. (2000). Human studies related to protein oxidation: protein carbonyl content as a marker of damage, *Free Radic Res*, **33**, suppl 99-108.
16. Mivata T., Sugiyama S., Suzuki D., Inagi R., Kurakowa K. (1999). Increased carbonyl modification by lipids and carbohydrates in diabetic nephropathy, *Kidney Int*, (**Suppl 71**), S54-56.

17. Raj D.S.J., Choudhury D., Welbourne T.C., Levi M. (2000). Advanced glycation end products: a nephrologist's perspective, *Am J Kidney Disease*, **35**, 365-380.

18. Soulis-Liparota T., Cooper M.E., Papazoglou D., Clarke B., Jerums G. (1991). Retardation by Aminoguanidine of development of albuminuria, mesengial expansion and tissue fluorescence in streptozotocin-induced diabetic rat, *Diabetes*, **40**, 1328-1334.

19. Kikuchi K., Nagano T., Haiakawa Hirata Y., Hirote M. (1993). Real time measurement of nitric oxide produced ex vivo by luminol-H2O2 chemiluminescence method. *J Biol Chem*, **268**, 23106-23110.

20. Yalçın A.S., Haklar G., Küçükkaya B., Yüksel M. Dalaman G. (1998). Chemiluminesence measurements for the detection of free radical species. In *Free Radicals. Oxidative Stress and Antioxidants* edited by T. Ozben pp. 355-390. New York, Plenium Press.

21. Levine R.L., Garland D., Oliven C.N., Amici A., Climent I., Lenz A.G., Ahn B.V., Shantiel S., Stadtman E.R. (1990). Determination of carbonyl content in oxidatively modified proteins. *Methods Enzymol,* **186**, 464-478.

22. Kakkar R., Kalra J., Mantha S.V., Prasad K. (1995). Lipid peroxidation and activity of antioxidant enzymes in diabetic rats, *Moll Cell Biochem*, **151** (2),113-119.

23. Brounlich H., Marx F., and Stein G. (1994). Glutathion status, lipid peroxidation and kidney function in streptozotocin diabetic rats, *Exp Toxicol Pathol*, **46**(2), 143-7.

24. Suzuki D., Miyata T., Saotome N., Horie K., Inagi R., Yasuda Y., Uchida K., Izuhara Y., Yagama M., Sakai H. Kurokawa K. (1999). Immunohistochemical evidence for an increased oxidative stress and carbonyl modification of proteins in diabetic glomerular lesions. *J Am Soc Nephrol*, **10**, 822-832.

25. Brownlee M., Vlasara H., Kooney A., Ulrich P., Cerami A. (1986). Aminoguanidine prevents diabetes-induced arterial wall protein cross-linking. *Science,* **232**, 1629-1632.

26. Raij L., Baylis C. (1995). Glomerular actions of nitric oxide. *Kidney Int,* **48**, 20-32.

27. Keynan S., Hirsberg B., Levin-Iaina N., Wexler ID., Dahan R., Reinhartz E., Ovadia H., Wollman Y., Chernihovskey T., Iaina A., Raz I. (2000). Renal nitric oxide production during early phase of experimental diabetes mellitus. *Kidney Int*., **58**, 740-747.

28. Craven P.A., Struder R.K., DeRubertis F.R. (1995). Impaired nitric oxide release by glomeruli from diabetic rats, *Metabolism*, **44**, 695-698.

